# Analysis of the Isomerase and Chaperone-Like Activities of an Amebic PDI (*Eh*PDI)

**DOI:** 10.1155/2015/286972

**Published:** 2015-01-28

**Authors:** Rosa E. Mares, Alexis Z. Minchaca, Salvador Villagrana, Samuel G. Meléndez-López, Marco A. Ramos

**Affiliations:** Facultad de Ciencias Químicas e Ingeniería, Universidad Autónoma de Baja California, Calzada Universidad 14418, Parque Industrial Internacional, 22390 Tijuana, BCN, Mexico

## Abstract

Protein disulfide isomerases (PDI) are eukaryotic oxidoreductases that catalyze the formation and rearrangement of disulfide bonds during folding of substrate proteins. Structurally, PDI enzymes share as a common feature the presence of at least one active thioredoxin-like domain. PDI enzymes are also involved in holding, refolding, and degradation of unfolded or misfolded proteins during stressful conditions. The *Eh*PDI enzyme (a 38 kDa polypeptide with two active thioredoxin-like domains) has been used as a model to gain insights into protein folding and disulfide bond formation in *E. histolytica*. Here, we performed a functional complementation assay, using a Δ*dsb*C mutant of *E. coli*, to test whether *Eh*PDI exhibits isomerase activity *in vivo*. Our preliminary results showed that *Eh*PDI exhibits isomerase activity; however, further mutagenic analysis revealed significant differences in the functional role of each thioredoxin-like domain. Additional studies confirmed that *Eh*PDI protects heat-labile enzymes against thermal inactivation, extending our knowledge about its chaperone-like activity. The characterization of *Eh*PDI, as an oxidative folding catalyst with chaperone-like function, represents the initial step to dissect the molecular mechanisms involved in protein folding in *E. histolytica*.

## 1. Introduction

In eukaryotic cells, folding and posttranslational modifications of proteins are the primary function of the endoplasmic reticulum (ER) [1]. Formation of disulfide bonds, a common modification observed in several secretory proteins, takes place mainly in that compartment [1, 2]. Almost all organisms have a set of proteins involved in folding; however, the cellular and molecular details of this process have been elucidated only in a few model systems, such as the yeast ER and the bacterial periplasmic compartment [3, 4].

Protein disulfide isomerases (PDI) are eukaryotic oxidoreductases that catalyze the formation and rearrangement of disulfide bonds during folding of substrate proteins [5]. Structurally, PDI enzymes share as a common feature the presence of at least one active thioredoxin-like domain. Some organisms, such as yeast and mammals, have a family of PDI homologues that exhibit distinct domain organization and function [6–8].

Under physiological conditions, the cellular mechanisms that respond to proteotoxic stress remain in an inactive state; however, under stressful conditions, several response mechanisms are triggered to restore proteome stability, but if these fail, the apoptotic pathways are activated, leading ultimately to cell death [9, 10]. In addition to assisting oxidative folding of nascent polypeptides, PDI enzymes are also involved in holding, refolding, and degradation of unfolded or misfolded proteins under stressful conditions [10]. Furthermore, since the blocking of PDI activity could lead to protein misfolding, prolonged proteotoxic stress and apoptosis [11, 12] considering PDI as a therapeutic target to stop the progression of some diseases seem plausible [13, 14].

Human amebiasis, the parasitic infection caused by the protozoan* Entamoeba histolytica*, is a prevalent infection in developing countries [15]. Interestingly, the continued secretion of proteins, including virulence factors, is well recognized as the primary feature of this parasite [16]. Moreover, protein folding and correct disulfide bond formation are essential for secreted virulence factors, such as the Gal/GalNAc-inhibitable lectin [17] and the pore-forming peptide A (amoebapore A) [18].

The* E. histolytica* genome has 11 genes encoding PDI homologues [19]. From these, the enzyme named* Eh*PDI (a 38 kDa polypeptide with two active thioredoxin-like domains) has been used as a model to study protein folding and disulfide bond formation in* E. histolytica*. By using* in vitro* assays and standard substrates (such as insulin, lysozyme, and ribonuclease A), we have confirmed that* Eh*PDI exhibits the distinctive oxidoreductase activities (reductase, oxidase, and isomerase) as well as the typical chaperone-like function (suppression of polypeptide aggregation) [20, 21]. Only the oxidase activity has been demonstrated* in vivo*, through functional complementation of the* dsb*A mutation in* E. coli* [22].

Here, to test whether* Eh*PDI exhibits isomerase activity* in vivo*, we performed a functional complementation assay using the Δ*dsb*C mutant of* E. coli* as a model and the defective expression of the periplasmic protein AppA as the phenotype. The acid phosphatase-phytase enzyme (AppA) has three consecutive disulfide bonds and one nonconsecutive that renders it dependent on DsbC [23]. Our preliminary results showed that* Eh*PDI exhibits isomerase activity; however, further mutagenic analysis revealed significant differences in the functional role of each thioredoxin-like domain. Finally, additional studies confirmed that* Eh*PDI protects two heat-labile enzymes, *α*-glucosidase and* Nde*I endonuclease, against thermal inactivation, extending our knowledge about its chaperone-like activity.

## 2. Materials and Methods

### 2.1. Materials

DNA amplification reagents and DNA purification kits were from Qiagen (Valencia, CA). Bacterial media were from Becton Dickinson (Franklin Lakes, NJ). Electrophoresis reagents were from Bio-Rad (Hercules, CA). Endonucleases and other enzymes were from New England Biolabs (Ipswich, MA). Other biochemicals were from Sigma-Aldrich (St. Louis, MO), otherwise mentioned in the text. All reagents used were analytical or molecular biology grade.

### 2.2. Bacterial Strains, Plasmids, and Growth Conditions

*Escherichia coli* strains and plasmids used in this study are listed in Table 1. Bacterial cultures were grown in LB medium at 37°C, with appropriate antibiotics (ampicillin at 150 *μ*g/mL and chloramphenicol at 15 *μ*g/mL). Recombinant plasmids were constructed by using standard molecular cloning protocols.

#### 2.2.1. Construction of pBAD-AppA Plasmid

Full-length sequence of the bacterial* app*A gene was amplified from genomic DNA (XL1-Blue MRF′ strain), using the synthetic oligonucleotides EcAppAF (5′-cgc gcg gaa ttc ATG AAA AGC GGA AAC ATA TCG-3′) and EcAppAR (5′-cgc gcg tct aga TTA CAA ACT GCA CGC CGG TAT-3′) as primers. The PCR product was then digested with* Eco*RI and* Xba*I endonucleases and cloned into the* Eco*RI-*Xba*I sites of pBluescript SK-, yielding the pBAppA plasmid. To obtain the pBAD-AppA plasmid, a site for* Eco*RV (located immediately upstream of the* Eco*RI) was used to get a restriction fragment from* Eco*RV-*Xba*I sites, which was then subcloned into* Sma*I-*Xba*I sites of pBAD33. The* app*A gene was confirmed by DNA sequencing.

#### 2.2.2. Construction of the pQRM05, pQRM06, and pQRM15 Plasmids

The* Eh*PDI gene variants (with Cys to Ser substitutions) were amplified from its corresponding pBluescript-based plasmid (Table 1), using the synthetic oligonucleotides* Eh*PDIp38F (5′-cat cac gga tcc GCT GAT GTA GTA TCA TTA AAT C-3′) and M13FW (5′-GTA AAA CGA CGG CCA GTG-3′) as primers. Then, PCR products were digested with* Bam*HI and* Hin*dIII endonucleases and subcloned into the same sites of pQE30 (in frame with the sequence encoding the N-terminal hexahistidine tag). The* Eh*PDI gene variants were confirmed by DNA sequencing.

### 2.3. DsbC Complementation and AppA Activity Assay

#### 2.3.1. Periplasmic Expression of AppA and Coexpression with* Eh*PDI

*E. coli* strains BW25113 (wild type) and JW2861-1 (Δ*dsb*C mutant) were transformed with pBAD33 (as control) or pBAD-AppA. Stable transformants were cultured in LB medium, supplemented with chloramphenicol, and the periplasmic expression of AppA was induced with 0.2% arabinose. Bacterial cell pellets (from 1 mL) were obtained by centrifugation (2 min at 10,000 rpm).

*E. coli* strain JW2861-1 harboring pBAD-AppA was transformed with pBluescript-based plasmids expressing* Eh*PDI variants (Table 1). The plasmid pBluescript SK- was used as a control. Stable cotransformants were cultured in LB medium, supplemented with ampicillin and chloramphenicol, and the periplasmic coexpression of AppA and* Eh*PDI was induced with 0.2% arabinose and 1 mM IPTG. Bacterial cell pellets were obtained as before.

#### 2.3.2. Acid Phosphatase Activity Assay

The acid phosphatase activity was determined by a colorimetric assay [23]. Bacterial cell pellets were resuspended in glycine buffer (0.25 M; pH 2.5) and adjusted to 0.3–0.6 units of* A*_600_ per mL. Then, 20 *μ*L aliquots were further diluted with 80 *μ*L of the same buffer and mixed with 100 *μ*L of 50 mM* p*-nitrophenyl phosphate. After 15 min of incubation at 37°C, reactions were stopped by adding 1 mL of 1.2 N NaOH. Immediately, supernatants were separated by centrifugation (5 min at 14,500 rpm) and the released* p*-nitrophenolate was quantified by measuring the* A*_420_. Light scattering by cellular debris was also considered (recording the* A*_550_). The acid phosphatase activity was expressed in Miller units [23, 24].

### 2.4. Purification of Recombinant* Eh*PDI Enzymes

*E. coli* strain Shuffle Express was transformed with pQE30-based plasmids expressing recombinant* Eh*PDI enzyme variants (see Table 1). Stable transformants were cultured in LB medium, supplemented with ampicillin, and protein expression was induced with 0.1 mM IPTG. Bacterial cells (from 100 mL) were harvested and lysed under native conditions, using the CelLytic B Plus Kit (Sigma-Aldrich). From the soluble fraction, recombinant proteins were purified by Ni-affinity chromatography (The QIAexpressionist, Qiagen). Eluate fractions were analyzed by SDS-PAGE and those containing more than 95% of pure protein were pooled and concentrated/desalted by ultrafiltration, using a Microsep UF Spin Filter (Pall Co.). Protein concentration was determined by performing the BCA colorimetric assay (Sigma-Aldrich), using BSA as standard.

### 2.5. Oxidative Refolding Assay

Oxidative refolding of denatured-reduced ribonuclease A (drRNAse) by recombinant amebic PDI enzymes was assayed by following a reported protocol [25]. Refolding was achieved by diluting drRNAse (7.8 *μ*M) into a reaction buffer (2 mM GSH, 0.4 mM GSSG, 100 mm Tris-HCl, pH 8.0) containing 5 *μ*M of amebic PDI enzymes and 4.5 mM of cCMP (RNAse substrate). The reactivation of RNAse was followed for 60 min by recording the absorbance at 296 nm. Active RNAse (*μ*M) was calculated from the first derivative of the absorbance over time and corrected for the depletion of the substrate and the formation of the product (CMP, RNAse inhibitor) [25]. The isomerase activity was determined from the linear increase of active RNAse over time (*μ*M/min), after the lag phase (which reflects the oxidase activity, min^−1^).

### 2.6. Disulfide Reductase Assay

Disulfide reduction of bovine insulin catalyzed by recombinant amebic PDI enzymes was assayed according to a standard turbidity method [21, 26]. Recombinant enzymes (2 *μ*M final) were added to a reaction buffer (2 mM EDTA and 100 mM HEPES; pH 7.0) containing bovine insulin (100 *μ*M final). Disulfide reduction was started by adding DTT (0.3 mM final) and followed for 90 min by recording the* A*_650_ every 5 min. Reductase activity was determined from the linear increase of absorbance over time after the lag phase (*A*_650_/min^2^) [27].

### 2.7. Chaperone Activity Assays

Chaperone-like activity of* Eh*PDI was evaluated by performing a protection against thermal inactivation assay of two heat-labile enzymes: *α*-glucosidase [28, 29] and* Nde*I endonuclease [30, 31].

#### 2.7.1. Thermal Inactivation of *α*-Glucosidase

Different concentrations of* Eh*PDI (0–5 *μ*M final) were added to a reaction buffer (50 mM KH_2_PO_4_; pH 6.8) containing yeast *α*-glucosidase (16 *μ*g/mL final). Thermal inactivation was performed by incubating at 43°C for 60 min (a control without treatment was carried out for each concentration). Then, reactions were cooled on ice for 1 min and the aggregated protein was separated by centrifugation (5 min at 14,500 rpm). *α*-glucosidase activity was determined by diluting a 40 *μ*L aliquot of the supernatant with 160 *μ*L of reaction buffer containing 0.125 mM of reduced glutathione and 1.25 mM of* p*-nitrophenyl-*α*-D-glucopyranoside. After 20 min of incubation at room temperature, reactions were stopped by adding 50 *μ*L of 0.5 M Na_2_CO_3_. The released* p*-nitrophenolate was quantified by measuring the absorbance at 415 nm. The *α*-glucosidase activity (*AG*) was defined by the increase in absorbance over time (*A*_415_/min). For each concentration of* Eh*PDI, the percentage of protection against thermal inactivation (chaperone-like activity) was determined by using the following equation: protection (%) = (*AG*_T_/*AG*_U_) × 100, where *AG*_T_ represents the remaining *α*-glucosidase activity after thermal treatment (60 min at 43°C), while the untreated enzyme is represented by *AG*_U_.

#### 2.7.2. Thermal Inactivation of* Nde*I Endonuclease

Different concentrations of* Eh*PDI (0–2 *μ*M final) were added to a reaction buffer (75 mM potassium acetate, 30 mM Tris-acetate, 15 mM magnesium acetate, 1.5 mM DTT; pH 7.9) containing* Nde*I endonuclease (1 U/*μ*L final). Thermal inactivation was performed by incubating at 50°C for 30 min (control reactions without incubation were carried out). Then, the reactions were cooled on ice for 2 min and briefly centrifuged (to collect a 10 *μ*L volume). Endonucleolytic activity was determined by adding 5 *μ*L of a plasmid solution, containing 0.1 *μ*g of pUC19 and 0.1 mg of BSA. After 2 hours of incubation at 37°C, standard agarose gel electrophoresis and ethidium bromide staining were used to analyze the restriction fragments. The* Nde*I endonuclease activity was defined by the relative amount of linearized plasmid, estimated by digital densitometry. For each concentration of* Eh*PDI, the percentage of protection against thermal inactivation (chaperone-like activity) was determined by using the following equation: protection (%) = [(*EN*_T_ − C2)/(C1 − C2)] × 100, where *EN*_T_ represents the remaining* Nde*I endonuclease activity after thermal treatment (30 min at 50°C), while C1 (with* Nde*I) and C2 (without* Nde*I) correspond to the control reactions without treatment.

### 2.8. Statistical Analysis

Unless otherwise mentioned, activity data were from three independent experiments and are represented as mean ± standard error. All statistical analysis were performed using Prism v.5 (GraphPad Software, San Diego, CA). Unpaired* t*-test was used for routine comparison of data sets. *P* values less than 0.05 were considered statistically significant.

## 3. Results and Discussion

### 3.1. *Eh*PDI Exhibits* In Vivo* Isomerase Activity

To study the functional activities of eukaryotic PDI enzymes* in vivo*, yeast and bacterial cells have been successfully used to complement phenotypes associated with defective formation of disulfide bonds [32, 33]. In* E. coli* cells, the oxidative folding of polypeptides is carried out in the periplasmic compartment and performed by the Dsb proteins: oxidation and isomerization of disulfide bonds are catalyzed by DsbA and DsbC, respectively [34].

The DsbC protein is particularly notable since it shares structural and functional similarities with eukaryotic PDI enzymes [35]. In fact, its functional role as disulfide isomerase has been studied using eukaryotic multidisulfide proteins as substrates [35, 36]. Four physiological substrates of DsbC have been identified so far: AppA [23], RcsF [37], MepA, and RNAse I [38]; from these,* in vivo* studies using AppA as substrate protein showed that DsbC plays an important role during folding of proteins with nonconsecutive disulfide bonds [23]. Then, to test whether* Eh*PDI exhibits disulfide isomerase activity* in vivo*, we performed a functional complementation assay using the Δ*dsb*C mutant of* E. coli* as a model and the defective periplasmic expression of AppA as the phenotype.

**Figure 1:**
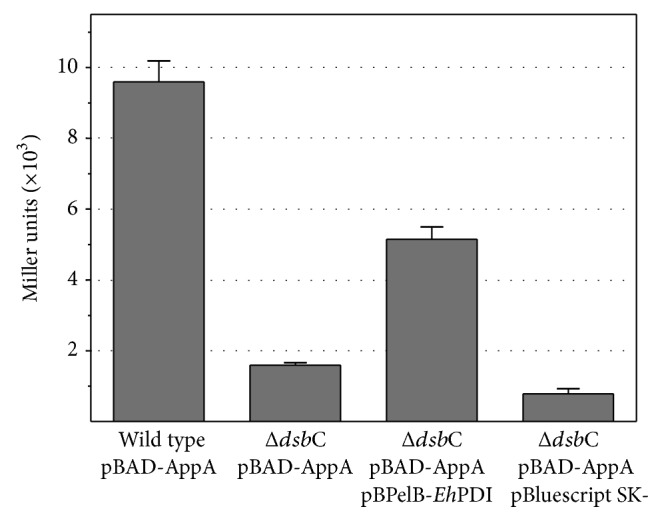
Acid phosphatase activity when AppA was expressed in the wild type and Δ*dsb*C mutant strains, as well as when it was coexpressed with* Eh*PDI in the Δ*dsb*C mutant strain. The activity (expressed in Miller units) is shown on the left. The plasmids used for transfection of* E. coli* are also indicated.

Initially, the absence of acid phosphatase activity (as background) was confirmed, indicating that the chromosomal* app*A gene was not induced under our experimental conditions (data not shown). Then, pBAD-AppA (Table 1) was used to transform both wild type and Δ*dsb*C mutant strains. Stable transformants were cultured and AppA expression was induced properly. As noted in Figure 1, a high level of acid phosphatase activity was detected in the wild type strain, 9595 ± 593 Miller units, whereas a low level of activity was observed in the Δ*dsb*C mutant (1593 ± 70 Miller units), confirming the DsbC-dependence of AppA (Figure 1). Then, pBPelB-*Eh*PDI (Table 1) was used to transform the Δ*dsb*C/pBAD-AppA strain, using a two-plasmid system [39]. Stable cotransformants were cultured and protein expression was induced accordingly. Periplasmic expression of* Eh*PDI was confirmed by immunoblot (data not shown). Interestingly, a significant increase in acid phosphatase activity was detected (5151 ± 344 Miller units), suggesting that the correct disulfide bond formation of AppA was assisted by the isomerase activity of* Eh*PDI.

### 3.2. Isomerase Activity of* Eh*PDI Is Limited and Dependent on Its Reductase Activity

The active site of the thioredoxin-like domains from DsbA, DsbC, and PDI enzymes is characterized by the presence of the motif CXXC, where the cysteine residues play an important role in the enzymatic activity [40, 41].* Eh*PDI contains two thioredoxin-like domains (referred to as N- and C-Trx, resp.; both having the motif CGHC) that are essential for its* in vivo* oxidase activity [20]. To test whether both domains contribute to the isomerase activity, we carried out mutagenic analysis followed by a functional complementation assay.

The pBluescript-based plasmids expressing* Eh*PDI variants (Table 1) were used to transform the Δ*dsb*C/pBAD-AppA strain. Stable cotransformants were cultured and protein expression was induced accordingly. Periplasmic expression of* Eh*PDI variants was confirmed by immunoblot (data not shown). As indicated in Table 2, the isomerase activity of* Eh*PDI is dependent on its CGHC active sites, since a complete loss of the AppA activity was observed when the variant having both domains inactivated was coexpressed (*Eh*PDI_SS/SS_). Also, low AppA activity was detected when each domain was tested without the background of the other (*Eh*PDI_SS/CC_ and *Eh*PDI_CC/SS_); furthermore, the slight difference observed between these two variants can be explained by considering that the thioredoxin-like domains are not equivalent with regard to the isomerase activity [33, 42]. However, it is important to take into account the cellular features of the bacterial model to better understand the role of* Eh*PDI as an isomerase* in vivo*.

In the periplasmic compartment, substrate proteins with misoxidized disulfide bonds are shuffled to properly oxidized states by two mechanisms: (1) the isomerase pathway, where DsbC acts on the substrate as reductase-oxidase, and (2) the reductase pathway, where DsbC simply acts as reductase, allowing DsbA another chance to correctly oxidize the substrate [43]. In addition, when the Δ*dsb*C mutant of* E. coli* was complemented with the protein TrxP from* Bacteroides fragilis* (a periplasmic reductase with poor isomerase activity), a fully restored AppA activity was observed, indicating that the disulfide bond isomerization of this substrate is accomplished mainly through the reductase pathway [43]. Hence, it is reasonable to think that the low AppA activity detected when the Δ*dsb*C mutant of* E. coli* was complemented with any of the variants (Table 2) suggests that the isomerase function of* Eh*PDI is dependent on its reductase activity. To test this, we performed two* in vitro* activity assays, oxidative refolding of ribonuclease and reduction of insulin, using purified recombinant enzymes, that is,* Eh*PDI variants.

As indicated in Table 3, the wild type (*Eh*PDI) and variants (*Eh*PDI_SS/CC_ and *Eh*PDI_CC/SS_) showed comparable oxidative refolding capabilities. In contrast, significant differences were observed in their reductive activities: *Eh*PDI_CC/SS_ retained about 60%, whilst *Eh*PDI_SS/CC_ roughly retained 20%. These results confirmed that* Eh*PDI is dependent on its reductase activity to function as oxidoreductase* in vivo* and* in vitro*.

### 3.3. *Eh*PDI Protects Proteins against Thermal-Induced Aggregation

The chaperone-like function of PDI enzymes is determined by their ability to protect misfolded/unfolded substrate proteins against thermal-induced aggregation and to assist refolding [44, 45]. This function is essential in order for PDI to act as an efficient folding catalyst, since it allows access to buried thiols and disulfide bonds in the substrates and prevents nonspecific interactions between partially folded intermediates [1]. Typically, the chaperone-like function has been studied* in vitro* by measuring the ability to prevent protein aggregation induced by different physical or chemical conditions, such as temperature or denaturants [5, 46].

We have already reported that* Eh*PDI exhibits chaperone-like function by showing its ability to prevent the DTT-induced aggregation of the B chain of insulin [21]. Although this assay was a simple approach to test the chaperone-like function of* Eh*PDI, the low molecular mass of the substrate (3.4 kDa) represents a limitation of this assay, since it offers a restricted number of contact sites to form stable complexes [47]. Hence, to gain further insights regarding the chaperone-like function of* Eh*PDI, we performed additional* in vitro* assays to test its ability to prevent thermal-induced aggregation, using as substrates two heat-labile enzymes: *α*-glucosidase and* Nde*I endonuclease.

**Figure 2:**
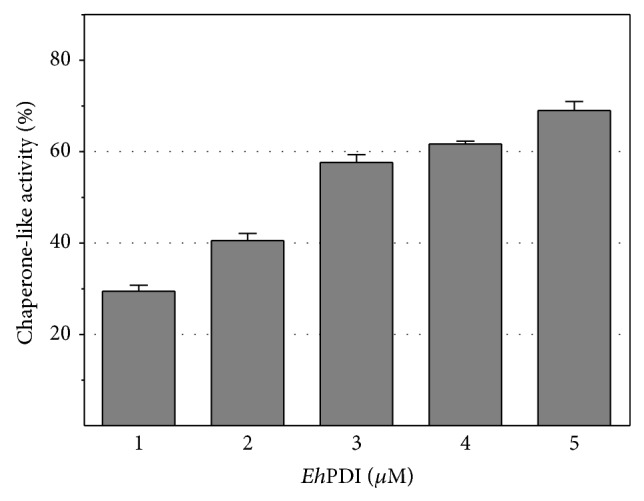
Protection of thermal inactivation of *α*-glucosidase assisted by* Eh*PDI. Relative chaperone-like activity (%) of recombinant* Eh*PDI.

**Figure 3:**
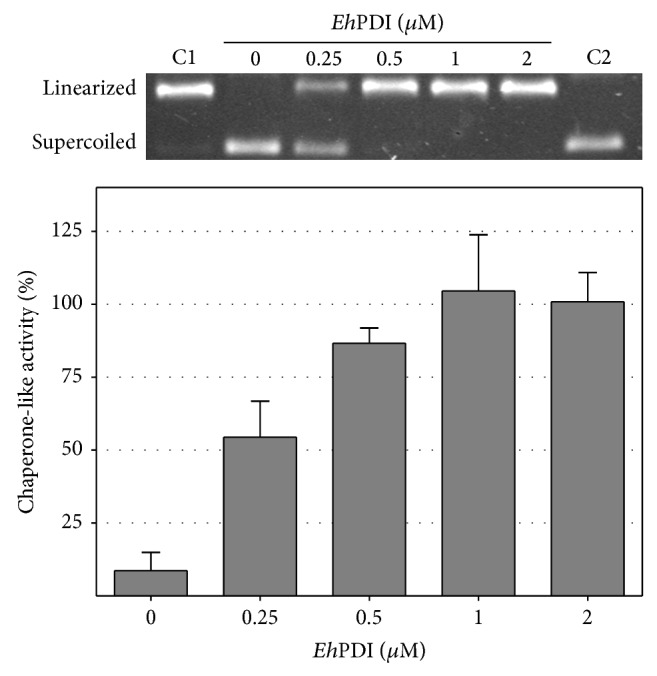
Protection of thermal inactivation of* Nde*I endonuclease assisted by* Eh*PDI. Relative chaperone-like activity (%) of recombinant* Eh*PDI. Upper panel: agarose gel indicating the relative mobility of the linearized and supercoiled plasmid (C1 and C2); also, concentrations of* Eh*PDI are indicated.

As shown in Figures 2 and 3, the chaperone-like function of* Eh*PDI was dose-dependent, since an increment of activity was observed as a result of augmenting its concentration. Moreover, to estimate its chaperone-like ability, the half-maximal effective concentration (EC_50_) and Hill slope were calculated by fitting the data to a model of one specific binding site with a variable slope. Interestingly, the apparent values obtained for *α*-glucosidase (EC_50_ = 3.0 ± 0.4 *μ*M; Hill slope = 1.0) and* Nde*I endonuclease (EC_50_ = 0.26 ± 0.06 *μ*M; Hill slope = 2.4) suggest that* Eh*PDI exhibits differences in substrate specificity and affinity [48].

Although* Eh*PDI does not have a substrate-binding b′-domain (a-a′-D) as the mammalian homologue (a-b-b′-a′-c) [49], the notion that some other domains (e.g., a′ or D) might be involved in the chaperone activity is evident. This idea is supported by the results of two previous reports: (i) ERp46, which lacks a b′-domain, is able to bind peptides through its catalytic domains (a^0^, a, and a′) [50] and (ii) the D-domain of ERp29 contains a discrete and conserved substrate-binding site [51].

### 3.4. Closing Remarks

Although little is known about the* E. histolytica* mechanisms that act in response to proteotoxic stress [52], the upregulation of genes encoding typical chaperones (such as Hsp70 and Hsp90) in a response to thermal stress suggests that it contains the cellular machinery necessary to preserve and restore the stability of the proteome [53]. So, the identification and characterization of* Eh*PDI as a folding catalyst with chaperone-like activity represents an additional step to dissect the molecular mechanisms involved in both protein folding and proteotoxic stress in* E. histolytica*. Hence, it is conceivable to suppose that inhibition of* Eh*PDI could lead to an increase in protein misfolding, promoting a sustained proteotoxic stress, eventually inducing apoptosis and, thus, preventing infection by this parasite.

## Figures and Tables

**Table 1:** Strains and plasmids used in this study.

Strains or plasmids	Relevant genotype or features	Source or reference
Strains		
XL1-Blue MRF′	*Δ *(*mcrA*)*183 Δ *(*mcrCB-hsdSMR-mrr*)*173 endA1 supE44 thi-1 recA1 gyrA96 relA1 lac *[*F*′* proAB lacI* ^*q*^ *ZΔM15 Tn10 *(*Tet* ^*R*^)]	Stratagene
Shuffle Express	*fhuA2 *[*lon*]* ompT ahpC gal *λ*att::pNEB3-r1-cDsbC *(*Spec* ^*R*^, *lacI* ^*q*^)*ΔtrxB sulA11 R *(*mcr-73::miniTn10– Tet* ^*S*^)*2 *[*dcm*]* R *(*zgb-210::Tn10– Tet* ^*S*^)* endA1 Δgor Δ *(*mcrC-mrr*)*114::IS10 *	NEB^1^
BW25113	*F* ^−^ * Δ *(*araD-araB*)*567 ΔlacZ4787*(::rrnB-3) *λ* ^−^ * rph-1 Δ *(*rhaD-rhaB*)*568 hsdR514 *	CGSC^2^ [54]
JW2861-1	BW25113* dsbC744::kan *	GCSC^2^ [54]
Plasmid		
pBAD33	Arabinose regulation, p15A origin, Cm^R^	ATCC^3^ [55]
pBAD-AppA	pBAD33-based, periplasmic AppA	This study
pBluescript SK-	Lactose regulation, ColE1 origin, Amp^R^	Stratagene
pBAppA	pBluescript-based, periplasmic AppA	This study
pBPelB-*Eh*PDI	pBluescript-based, periplasmic *Eh*PDI (wild type)	[22]
pBRM05	pBluescript-based, periplasmic* Eh*PDI_SS/CC_ (C44S; C47S)	[20]
pBRM06	pBluescript-based, periplasmic* Eh*PDI_CC/SS_ (C160S; C163S)	[20]
pBRM15	pBluescript-based, periplasmic* Eh*PDI_SS/SS_ (C44S; C47S; C160S; C163S)	[20]
pQE30	Lactose regulation, ColE1 origin, Amp^R^	Qiagen
pQHPDI	pQE30-based, recombinant *Eh*PDI (wild type)	[20]
pQRM05	pQE30-based, recombinant *Eh*PDI_SS/CC_ (C44S; C47S)	This study
pQRM06	pQE30-based, recombinant *Eh*PDI_CC/SS_ (C160S; C163S)	This study
pQRM15	pQE30-based, recombinant *Eh*PDI_SS/SS_ (C44S; C47S; C160S; C163S)	This study

^1^New England Biolabs; ^2^Coli Genetic Stock Center; ^3^American Type Culture Collection.

**Table 2:** *In vivo* isomerase activity of *Eh*PDI enzyme variants.

Enzyme variant	Acid phosphatase (AppA)^∗^ activity in Miller units (%)^¶^
*Eh*PDI_SS/SS_	732 ± 74 (−1)
*Eh*PDI_SS/CC_	1086 ± 136 (7)
*Eh*PDI_CC/SS_	1646 ± 230 (20)

^*∗*^The data was expressed as ± standard error (*n* = 6).

^¶^Under the background of Δ*dsb*C/pBAD-AppA, normalization was performed considering the mean activity of pBPelB-*Eh*PDI as maximal (*A*_max⁡_ = 5151) and pBluescript SK- as minimal (*A*_min⁡_ = 780). The percentage (%) was calculated as [(*A* − *A*_min⁡_)/(*A*_max⁡_ − *A*_min⁡_)] × 100, where *A* represents the activity of the enzyme variant.

**Table 3:** *In vitro* activities of purified *Eh*PDI enzyme variants.

Enzyme	RNAse A oxidative refolding^∗†¶^		Insulin reduction^∗†¶^ [×10^−6^ *A*_650_/min^2^] (%)
	Oxidase [×10^−6^ min^−1^] (%)	Isomerase [×10^−6^ μM/min] (%)	
*Eh*PDI	513 ± 16 (100)	39 ± 1 (100)	109 ± 11 (100)
*Eh*PDI_SS/CC_	551 ± 16 (107)	33 ± 1 (85)	22 ± 2 (20)
*Eh*PDI_CC/SS_	517 ± 13 (101)	31 ± 1 (79)	65 ± 5 (60)

^*∗*^The data was expressed as mean ± standard error (*n* = 3).

^†^The activity of the enzyme variant *Eh*PDI_SS/SS_ was not statistically significant, as compared with the reaction performed in the absence of enzyme.

^¶^The activity ratio (%) was calculated as (variant/wild type) × 100.
